# Discovery of novel hit compounds with broad activity against visceral and cutaneous *Leishmania* species by comparative phenotypic screening

**DOI:** 10.1038/s41598-018-36944-6

**Published:** 2019-01-24

**Authors:** S. Lamotte, N. Aulner, G. F. Späth, E. Prina

**Affiliations:** 1 Institut Pasteur, Molecular Parasitology and Signaling, INSERM U1201, Department of Parasites and Insect Vectors, Paris, France; 2 Institut Pasteur, UTechS Photonic BioImaging, Center for Technological Research and Resources, 75015 Paris, France

**Keywords:** Phenotypic screening, Parasitic infection

## Abstract

The limited success of recent phenotypic anti-leishmanial drug screening campaigns calls for new screening strategies for the discovery of clinically relevant hits. Here we present such a novel strategy based on physiologically relevant, *ex vivo* biology. We established high content phenotypic assays that combine primary murine macrophages and lesion-derived, virulent *L*. *donovani* and *L*. *amazonensis* amastigotes, which we applied to validate previously identified, anti-leishmanial hit compounds referred to as ‘GSK Leish-Box’. Together with secondary screens using cultured promastigotes, our pipeline distinguished stage- and/or species-specific compounds, including 20 hits with broad activity at 10 µM against intracellular amastigotes of both viscerotropic and dermotropic *Leishmania*. Even though the GSK Leish-Box hits were identified by phenotypic screening using THP-1 macrophage-like cells hosting culture-derived *L*. *donovani* LdBob parasites, our *ex vivo* assays only validated anti-leishmanial activity at 10 µM on intra-macrophagic *L*. *donovani* for 23 out of the 188 GSK Leish-Box hits. In conclusion, our comparative approach allowed the identification of hits with broad anti-leishmanial activity that represent interesting novel candidates to be tested in animal models. Physiologically more relevant screening approaches such as described here may reduce the very high attrition rate observed during pre-clinical and clinical phases of the drug development process.

## Introduction

Leishmaniases are neglected diseases caused by protozoan parasites of the genus *Leishmania* that are transmitted by the bite of female Phlebotomine sandflies. Almost 1 billion people are at risk of infection in close to 100 endemic countries throughout the tropical and subtropical regions of Africa, Asia, the Mediterranean countries and South and Central America^1^. Leishmaniasis clinical manifestations range from self-healing cutaneous lesions with possible mucosal dissemination to severe visceral forms, causing death if untreated.

In the absence of efficient reservoir and vector control strategies, and of preventive or therapeutic human vaccines, the mainstay of current intervention strategy to limit the disease is chemotherapy. Leishmaniases remain the only trypanosomatid diseases for which therapeutics are largely based on repurposed drugs, including antifungal (amphotericin B), anticancer (miltefosine), antibiotic (paromomycin) and antimalarial (sitamaquine) molecules^2,3^. However, all current treatments are limited and show serious drawbacks, including high cost that are prohibitory for resource-limited countries, poor compliance due to constraining mode of administration^4^, poor safety with important adverse effects due to toxicity^5^, treatment failure and drug resistance^6^. Similarly, current efforts to overcome the drug resistance by developing drug combination therapies^7,8^ may fail in light of the identification of an *L*. *infantum* strain that gained resistance against antimony and amphotericin B^9^. Multi-drug resistance was also documented in Indian field isolates^10^, and was confirmed experimentally by the *in vitro* selection of *L*. *donovani* parasites showing resistance to different drug combinations and even cross-resistance to unrelated drugs^11^. Combination therapy therefore may be of only limited use, raising important concerns on the current policy to promote this strategy. This alarming situation and the small number of novel therapeutic molecules available in the drug discovery pipeline signal the urgent need for the discovery of novel candidate drugs and drug targets for Leishmaniases.

The creation of various private-public partnerships in the last decades dedicated to anti-parasitic drug discovery directly responds to this need. Unfortunately, the establishment of novel screening methodologies and the availability of large compound libraries for screening campaigns did not yet translate into new therapeutics, raising questions on the suitability of protocols and pipelines used. A common strategy for compound screening is the use of host cell-free parasites, *i*.*e*. promastigotes or axenic amastigote forms, which are easy to handle and inexpensive to grow, thus allowing for high-throughput (HT), automatized, micro-well plate-based screening^12^. However, the biological relevance of such assays may be called into question given (i) the extensive genetic instability and virulence attenuation observed in cultured parasites^13–15^, and (ii) the biological differences in for example infectivity between axenic and bona fide, tissue-derived amastigotes^16^. The physiological targets of chemotherapy are proliferating and intracellular amastigotes. Various screening campaigns tried to simulate this stage using axenic amastigote parasitesincubated with immortalized, macrophage-like cell lines^17,18^, thus falling short in terms of biological relevance and infectivity. These screening conditions may explain the limited success of these efforts to yield novel, validated hit compounds. Although current portfolios contain some new candidate leads, some of which have been identified using phenotypic screens, screening methodology still needs to be improved to correspond closer to the biology of clinical infection and to consider intra-species and inter-species variability in parasite tropism, drug susceptibility and intracellular infection.

Conceivably, screening systems that do not respect the physiology of *Leishmania* infection may be less predictive for *in vivo* drug efficacy causing a high attrition rate during pre-clinical or clinical phases of the drug discovery pipeline. To increase the number of clinically relevant hits, we propose here a new procedure using an *ex vivo* strategy and performing parallel, comparative screening campaigns with different *Leishmania* developmental stages and *Leishmania* species. Our assay combines i) biologically relevant phenotypic High Content Assays (HCA) based on primary macrophages infected with virulent, animal-derived amastigotes from dermotropic or viscerotropic *Leishmania* species, and ii) viability assays on promastigotes from both *Leishmania* species. We applied our procedure to evaluate 188 hit compounds previously identified in a phenotypic, high-throughput capable screen using THP-1 cells and the avirulent *L*. *donovani*-LdBob strain^18^. Our results uncovered important stage- and species-specific differences in compound efficacy, and validated only a few hits from the original screen, thus emphasizing the importance of the assay’s biology in hit discovery. Our protocol revealed five compounds with activity at 1 µM or below against both *L*. *donovani* and *L*. *amazonensis* intramacrophagic amastigotes. These hit compounds present similar scaffolds already known to exhibit anti-leishmanial activity, thereby validating our screening approach as a fertile new platform for the future discovery of pan-active, anti-leishmanial drug candidates.

## Results

### Development, validation and optimization of a physiologically relevant phenotypic assay for *Leishmania donovani*

**Figure 1:**
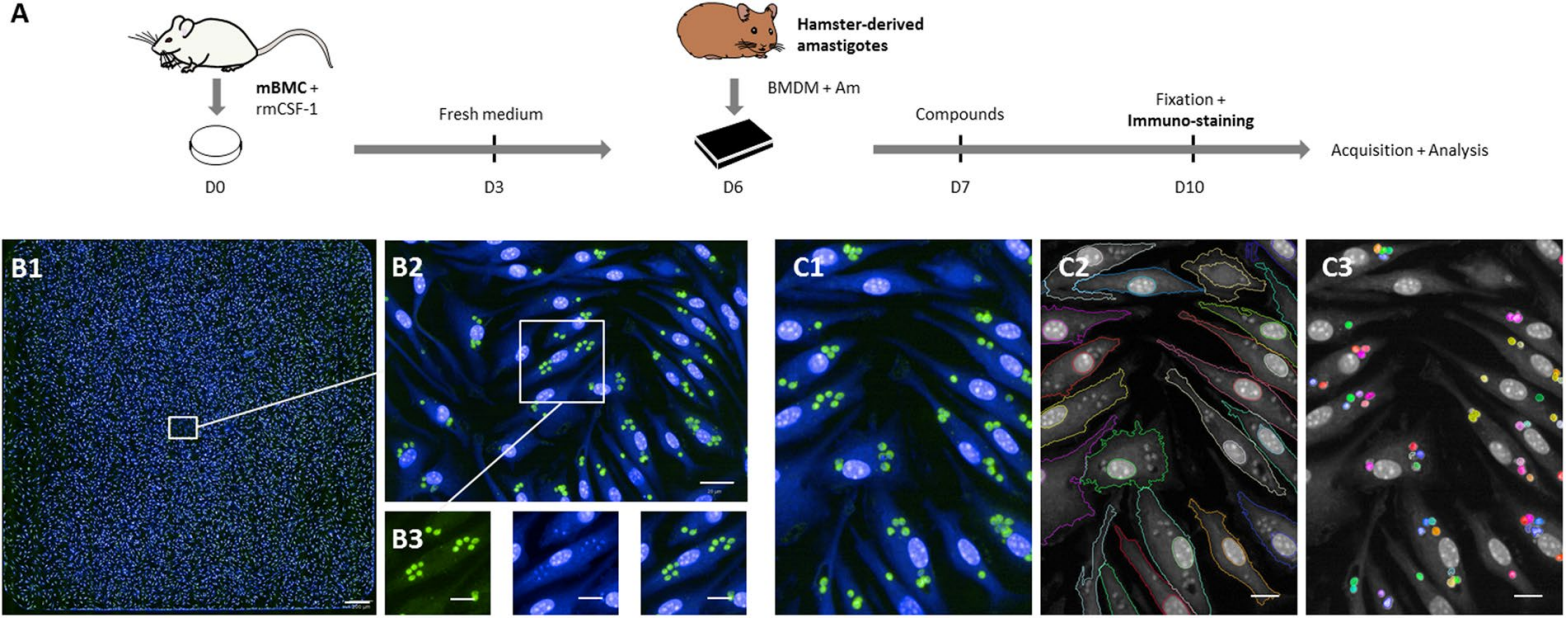
Schematic overview of the *L. donovani* screening assay. (**A**) High Content Assay (HCA) pipeline. Mouse bone marrow cells (mBMC) were cultured in presence of rmCSF-1 for 6 days before plating differentiated BMDMs in 384-well plates. Five and 20 hours later, hamster-derived amastigotes (Am) and compounds were added to BMDM cultures, respectively. After 3 days of incubation, BMDMs were fixed and subjected to immuno-staining followed by image acquisition and analysis. (**B1–3**) HCA readout. Image acquisition was performed with the OPERA QEHS High Content Imaging System using the following acquisition settings: 488 nm laser line for excitation and filter 540/75 for immuno-stained parasites; 405 nm laser line for excitation and filter 450/50 for Hoechst-stained nuclei and HCS CellMask cytoplasm detection. (**B1**) Merged fluorescence images acquired with a 40x water immersion objective (NA 1.5) showing the 285 fields of an entire well initially seeded with 20,000 BMDMs and inoculated with Ld1S amastigotes (MOI of 6). Scale bar, 200 µm. (**B2**) Zoomed image corresponding to one acquisition field. Scale bar, 20 µm. (**B3**) Cropped and zoomed image showing fluorescent staining at the cellular level. Left panel, immunostained amastigotes in green; middle panel, Hoechst 33342 and HCS CellMask staining in blue; right panel, merged images. Scale bars, 10 µm. (**C1–3**) Illustration of object segmentation pipeline implemented in the Columbus platform: initial overlay image (C1), macrophage cell and nucleus segmentation outline (C2) and intracellular parasites (C3). Scale bars, 10 µm.

Previous *L*. *donovani* screens were largely performed using either immortalized host cells and culture-adapted promastigote parasites or axenic amastigotes whose morphology, differentiation state, proteome and infectivity do not correspond to bona fide amastigotes^16^, which are the biologically relevant, ultimate target of chemotherapy. To increase the rate of hits targeting the clinically relevant, intracellular stage of *L*. *donovani*, we have developed a novel phenotypic High Content Assay (HCA) based on mouse primary macrophages that are infected with bona fide, tissue-derived *L*. *donovani* amastigotes. After 6 days of CSF-1-dependent differentiation from bone marrow progenitors, bone marrow-derived macrophages (BMDM) were collected and seeded into 384-well plates, then incubated with amastigotes that were purified from hamster-infected tissue. After a few hours, no extracellular amastigotes could be detected and the percentage of infected BMDMs remained constant during the 3-day HCA in control conditions without compound (data not shown). Twenty-four hours PI, compounds and controls - including DMSO vehicle - were added for a 3 days incubation period before fixation and immuno-staining, followed by image acquisition and analysis (Fig. 1A). Under these experimental conditions, amastigotes showed robust intramacrophagic proliferation^16^ inside LAMP1^+^ parasitophorous vacuoles (PV) that can be tracked and quantified during drug treatment (Fig. S1). In contrast to a previous HCA we established for *L*. *amazonensis*^19^ (Fig. S2) and for which the main readout of infected macrophages was the presence of large communal PVs stained with a fluorescent acidotropic probe, the *L*. *donovani* HCA readout was strictly dependent upon the number of individual, immuno-stained parasites. As a consequence, image acquisition was carried out at 40x magnification compared to 10x magnification used for the *L*. *amazonensis* HCA (Fig. 1B1). Applying an optimized, Columbus-embedded segmentation script on immunofluorescence images of fixed cells, we visualized and quantified (i) macrophages using the cytoplasmic staining HCS CellMask^TM^, (ii) macrophage and parasite nuclei using the nuclear dye Hoechst 33342, and (iii) intracellular parasites stained with an anti-*Leishmania* immune serum (Figs 1B2–1C3, S3). These readouts allowed automated assessment i) of compound toxicity by monitoring the number of host cells in comparison to DMSO controls, and ii) of compound anti-leishmanial activity by monitoring the number of amastigotes per macrophage, with activity being expressed as percentage of amastigote burden. A combination of both readouts classified compounds into active, weakly active, inactive, or toxic (Fig. S4).

### Validation of optimized HCA

**Figure 2:**
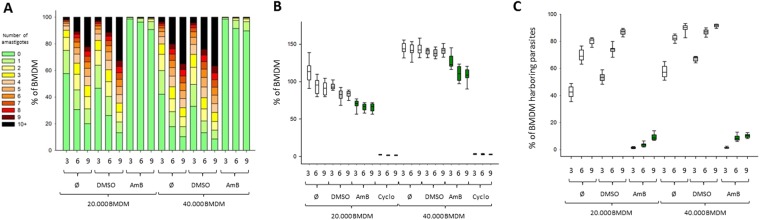
Validation and optimization of the *L. donovani* assay for compound screening. (**A**) Heatmaps of the distribution of BMDM according to the amount of intracellular amastigotes for each experimental condition, for different macrophage seeding densities (20,000 or 40,000 cells per well), MOIs (1:3, 1:6 or 1:9 macrophage to parasite ratio), and treatments (mock [Ø], DMSO and Amphotericin B [AmB]). (**B**) Boxplots of percentages of BMDMs normalized to mock control values obtained with 20,000 and 40,000 seeded macrophages and recovered after three days of treatment with DMSO (vehicle control), AmB (positive control) and cycloheximide (Cyclo, toxicity control) are represented. (**C**) Boxplots of percentages of BMDMs harboring parasites after 3 days for all experimental conditions except for cycloheximide. Whisker plot represents median and 5^th^/95^th^ percentiles.

We first optimized our assay with respect to the number of BMDMs plated per well, the number of parasites per BMDM, and the number of acquisition fields. As expected, cell density and MOI had a strong impact on infection efficiency, which ranged from 42.4% of infected macrophages containing an average of 2.8 parasites per cell (MOI of 3:1, 20,000 cells per well) to 89.7% of infected macrophages averaging 9.7 parasites per cell (MOI of 9:1, 40,000 cells per well) (Fig. 2A). While both AmB and DMSO vehicle showed some toxicity toward the host cell (Fig. 2B), changes in the MOI had a clear impact on drug efficacy, with a higher parasite clearance observed with AmB in macrophages infected with an MOI of 1:3 or 1:6 compared to 1:9 (Fig. 2C). Finally, we showed that reducing the number of acquisition fields from 285, representing an entire well, to 30 did not have an impact on parasite (Fig. S5A) and macrophage (Fig. S5B) quantification, with a strong correlation of parasite burden observed between both conditions (Fig. S5C). Thus, limiting the image acquisition step to 30 fields significantly accelerates the screening procedure without challenging its robustness. In conclusion, we used a seeding density of 20,000 macrophages per well, a MOI of 6:1, and an image acquisition of 30 fields per well as optimized settings to assess further the anti-leishmanial activity of Leish-Box compounds.

### Anti-leishmanial activity of Leish-Box compounds on intramacrophagic amastigotes

**Figure 3:**
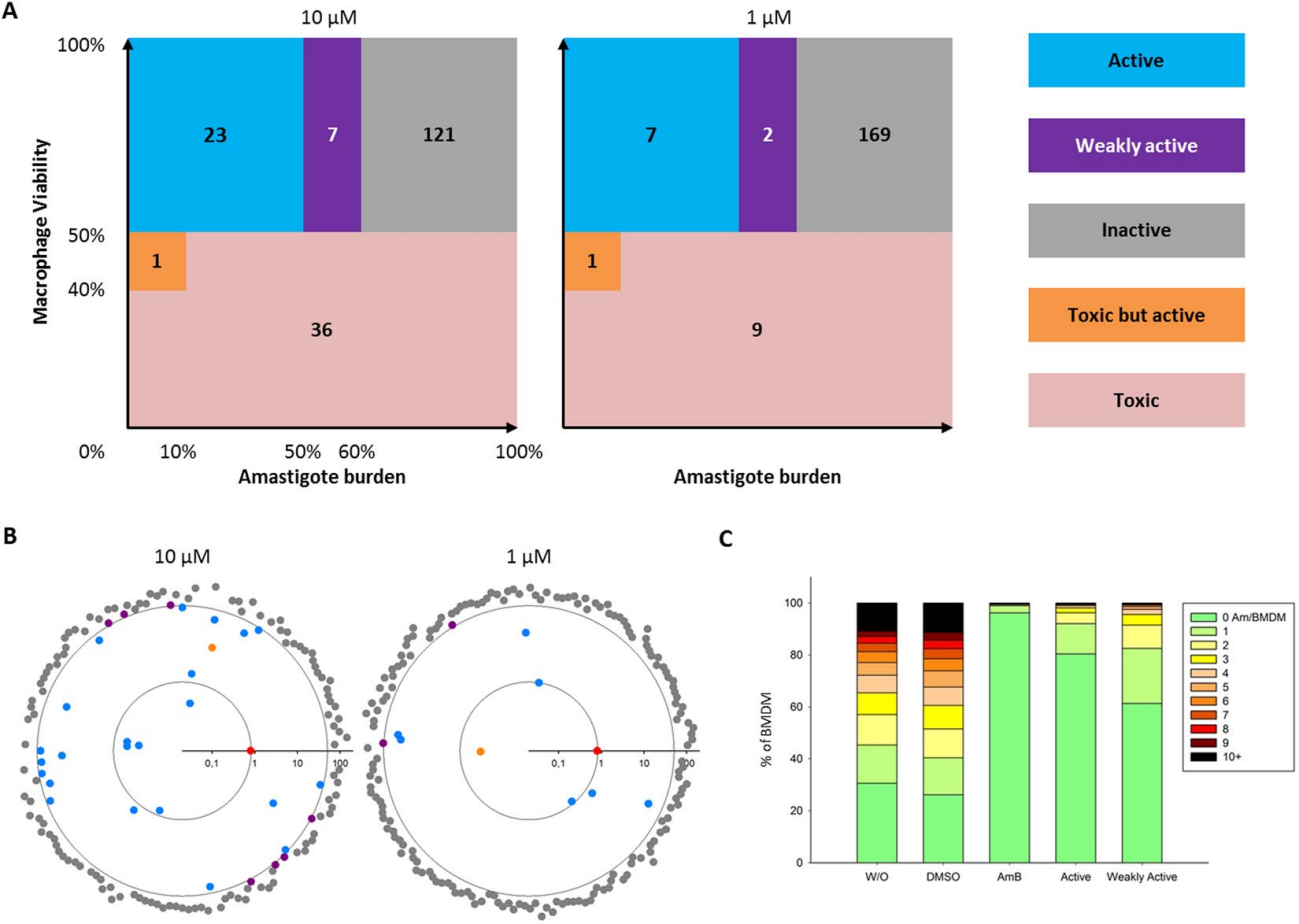
Leish-Box screening results for intracellular *L. donovani* amastigotes. Leish-Box compounds were tested at 10 µM (A and B, left panels) and 1 µM (A and B, right panels). All 188 compounds were classified according to defined categories. Anti-leishmanial activity is represented as Log10 of mean percentages of amastigotes survival compared to DMSO control as determined in quadruplicate experiments. Inactive, grey; weakly active, purple; active-toxic, orange; active, blue; anti-leishmanial positive control AmB (0.8% survival), red. The outer circle delineates 50% parasite survival and the inner circle delineates the 0.8% survival rate observed for AmB to emphasize very active compounds. Toxic compounds were excluded from this graph (35 and 9 compounds, at 10 and 1 µM, respectively, see Table 1). (**C**) Stacked bar plot displaying the level of infection observed under different culture conditions denoted on the x-axis. BMDMs were cultured without vehicle (w/o), in presence of DMSO or amphotericin B (AmB). The mean of all 23 active and 7 weakly active compounds at 10 µM is represented.

In 2015, GlaxoSmithKline (GSK) carried out an HT screening on 1.8 million compounds combining a primary phenotypic screen using cultured, axenic amastigotes of the experimental lab strain *L*. *donovani* LdBob (MHOM/SD/62/1S-CL2D, LdBob)^20^, and a secondary phenotypic screen using LdBob-infected THP-1 cells. This screening has identified 192 compounds with antileishmanial activity that are referred to as the “Leish-Box”^18^. We applied our improved, *ex vivo* assay to assess anti-leishmanial activity of 188 of these compounds (Table S1) on virulent, tissue-derived *L*. *donovani* amastigotes in primary mouse macrophages. As suspected from the biological differences between the GSK *in vitro* procedure and our *ex vivo* assays, the vast majority of Leish-Box compounds were inactive in our system when used at 1 µM final concentration but also at 10 µM (Fig. 3A, grey zone). POC values, i.e. Percent Of Control values, representing the parasite viability compared to DMSO control for these compounds were all above 60% (Fig. 3B, grey symbols). Only 23 and 7 compounds demonstrated strong anti-leishmanial activity (active compounds) at 10 and 1 µM, respectively, with POC values below 50% (Fig. 3B, blue symbols). These compounds caused a reduction of parasite burden similar to the AmB positive control (Fig. 3B, red symbol, and Fig. 3C). Seven and two compounds showed weak activity at 10 and 1 µM, respectively, with POC values between 50 and 60% (Fig. 3B, purple symbols), which nevertheless cleared infection in a majority of cells (Fig. 3C). A small number of compounds (one at 10 µM and one at 1 µM) were quite toxic for the host cells but still considered active as they strongly reduce the number of intracellular parasites in remaining living macrophages (Fig. 3B, orange symbols). Finally, 36 and 9 compounds were highly toxic for the macrophages at 10 and 1 µM, respectively, causing loss of more than 50% of the macrophages compared to the DMSO controls. These results illustrate that the physiology of the assay has a major impact on compound action, with observed differences in host cell toxicity and anti-leishmanial activity between assays likely caused either by differences in host cell or parasite biology. Alternatively, differences in host cell numbers, parasite MOI, incubation time with compounds, magnification, numbers of analyzed cells, and biological activity threshold used for compound classification may contribute to the differences observed between assays.

### Anti-leishmanial activity of Leish-Box compounds on cultured promastigotes

**Figure 4:**
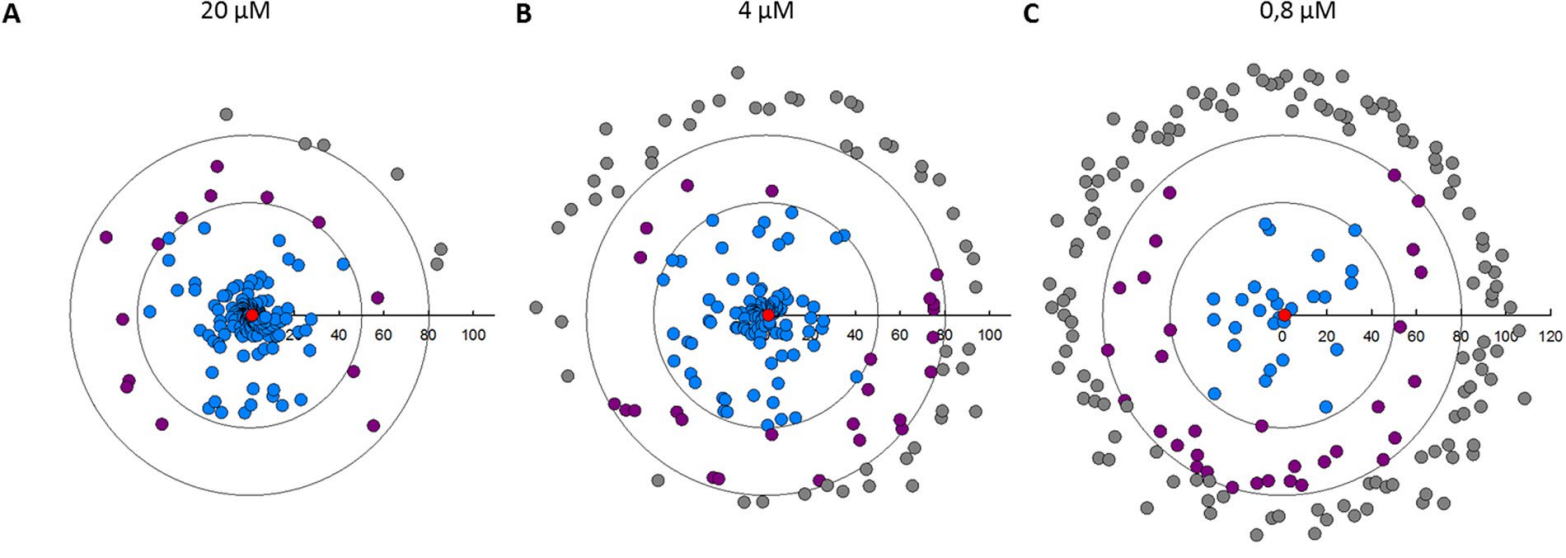
Leish-Box screening results for cultured *L. donovani* promastigotes. A resazurin-based viability assay was used to assess anti-leishmanial activity of Leish-Box compounds against Ld1S promastigotes. Two days after compound addition to parasite cultures at logarithmic growth phase, resazurin was added for an additional 24 hours before reading the level of resofurin fluorescence using an Infinite™ M200 plate reader (Tecan™). Anti-leishmanial activity of compounds are expressed as percent of untreated control (POC) of promastigote survival and displayed in circular plots for compound concentrations of 20 µM (**A**), 4 µM (**B**) and 0.8 µM (**C**). Inactive compounds (POC > 80%), grey; weakly active compounds (50 < POC < 80), purple; active compounds (POC < 50%), blue; AmB, red. The outer and inner circles represent respectively 80% and 50% threshold activity values.

For determining stage-specific activity, we next tested the Leish-Box compounds against *L*. *donovani* promastigotes in culture using a resazurin reduction assay to monitor parasite viability as a correlate for anti-leishmanial activity. At 20 µM, 168 compounds showed a strong anti-leishmanial activity with POC values lower than 50% (Fig. 4A, blue symbols). Only 6 compounds did not show any effect and 14 compounds showed a weak activity on promastigote viability (Fig. 4A, grey and purple symbols, respectively). These proportions were progressively inverted when compounds were tested at 4 and 0.8 µM, revealing respectively 109 and 27 compounds with strong activity (Fig. 4A–C, Table 1). These included 16 compounds showing an EC50 below 1 µM (data not shown), with the compound TCMDC143295 presenting the best anti-leishmanial activity with an EC50 of 80 nM. In conclusion, from the 188 compounds tested, 134 compounds showed anti-leishmanial activity against *L*. *donovani* promastigotes in the micromolar range. These data suggest that the original screen may have targeted extracellular parasites or poorly differentiated intracellular parasites rather than fully differentiated intracellular and cell-cycling amastigotes, for which a different susceptibility to drugs has otherwise been demonstrated^21,22^.

**Table 1:** Anti-leishmanial activity of Leish-Box compounds on cultured promastigotes using promastigote viability assay. Compounds have been classified according to their anti-leishmanial activity against *L. donovani* promastigotes expressed as percentages of untreated parasites (POC values).

	Active	Weakly Active	Inactive
	POC < 50%	50% < POC < 80%	POC > 80%
20 µM	168	14	6
4 µM	109	25	54
0,8 µM	27	32	129

### Anti-leishmanial Leish-Box compounds show stage- and species-specific activity

**Figure 5:**
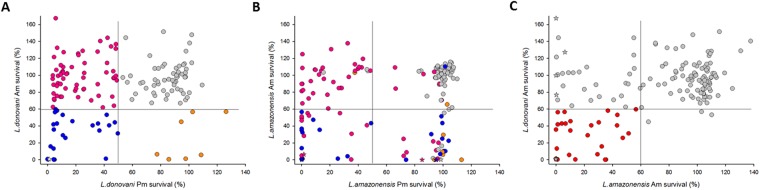
Stage- and species-specific activity of Leish-Box compounds. (**A**,**B**) Biparametric dot plots showing the survival of *L*. *donovani* parasites (**A**) and *L*. *amazonensis* parasites (**B**) after three days of treatment at 10 µM for intracellular amastigotes and 4 µM for cultured promastigotes. Results are expressed as percentages of parasite viability compared to DMSO control. Only compounds that were not toxic for BMDMs are represented. Inactive compounds, grey; active only on promastigotes, pink; active only on amastigotes, orange; active on both stages, blue, the color coding correspond to the activity on *L*. *donovani*. AmB activity is represented by the white star (panel A), and compounds showing toxicity on *L*. *amazonensis* infected macrophages are represented as colored stars (panel B). (**C**) Biparametric dot plot showing the survival of *L*. *donovani* and *L*. *amazonensis* amastigotes after three days of treatment at 10 µM. Results are expressed as percentages of parasite viability compared to DMSO control. Only compounds that were not toxic for *L*. *donovani* are represented. Compounds with activity on both species are indicated in red. Stars indicate compounds that were specifically toxic on *L*. *amazonensis* infected macrophages.

**Figure 6:**
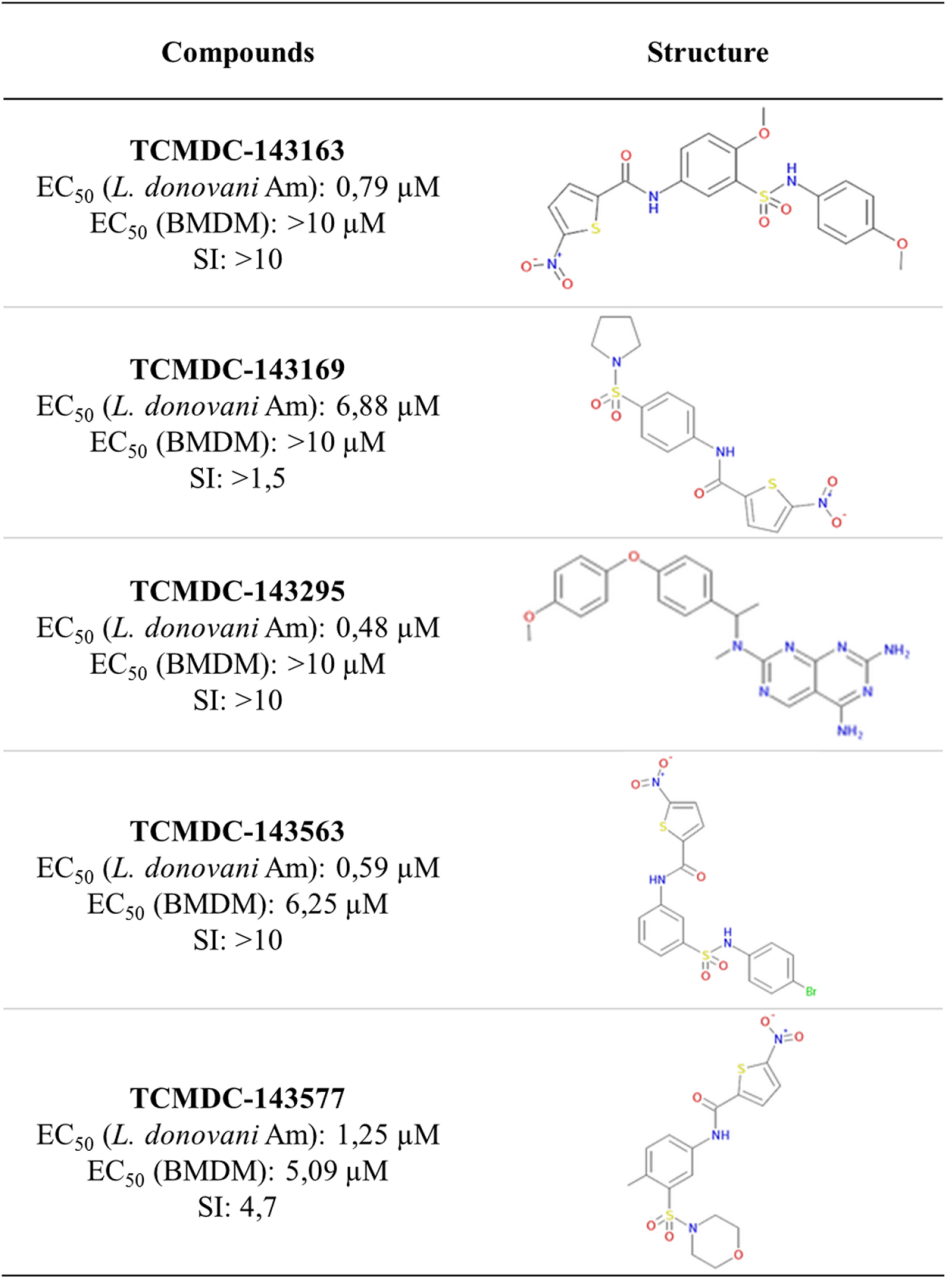
Hit compounds active on both *L*. *donovani* and *L*. *amazonensis* intramacrophagic amastigotes. EC50s for anti-parasitic activity against *L*. *donovani* and toxicity against macrophages, selectivity index and chemical structure are shown.

Comparison of the phenotypic screening results obtained with intracellular amastigotes and cultured promastigotes of *L. donovani* allowed us to distinguish four categories of compounds (Fig. 5A): i) inactive in both assays (59 compounds, grey symbols), ii) toxic for promastigotes but inactive against intramacrophagic amastigotes (62 compounds, pink symbols), iii) active only on intramacrophagic amastigotes (7 compounds, orange symbols), and iv) active on both *Leishmania* developmental stages (24 compounds, blue symbols). A similar pattern was obtained with the dermotropic *L*. *amazonensis* strain (Fig. 5B). Additionally, 5 and 3 compounds that were inactive against both *L*. *donovani* developmental stages displayed anti-leishmanial activity against *L*. *amazonensis* intramacrophagic amastigotes or promastigotes, respectively. Conversely, 10 active compounds against both stages of *L*. *donovani* (Fig. 5A, blue circles) were only active against *L*. *amazonensis* promastigotes (Fig. 5B, right quarters). Comparing compounds showing activity at 10 µM against either *L*. *donovani* or *L*. *amazonensis* amastigotes further allowed us to distinguish species-specific from broadly active compounds, revealing 28 compounds that target intramacrophagic amastigotes of both dermotropic and viscerotropic *Leishmania* species (Fig. 5C, lower left quarter, Fig. S6). Only three compounds with weak activity against *L*. *donovani* amastigotes were not active on *L*. *amazonensis* amastigotes (lower right quarter). Twenty-five compounds that were inactive against *L*. *donovani* amastigotes efficiently killed intramacrophagic *L*. *amazonensis* (upper left quarter). Finally, 88 compounds were considered inactive against amastigotes of both *Leishmania* species (upper right region). Only five compounds showed anti-leishmanial activity at the micromolar range on both *Leishmania* species with EC_50_ values in *L*. *donovani* HCA ranging between 0.48 µM and 6.88 µM (Fig. 6), emphasizing the importance of using physiologically relevant screening systems and parasite strains for the discovery of broadly active, anti-leishmanial hits.

## Discussion

Here we present a novel phenotypic screening strategy that relies on *ex vivo* biology, using bone marrow-derived macrophages infected with lesion-derived amastigotes of old world, viscerotropic *Leishmania donovani* Ld1S or new world, dermotropic *Leishmania amazonensis* LV79 strains. We applied these systems to validate the previously established Leish-Box hit library, representing 188 compounds that showed activity at 5 µM in the human macrophage-like cell line THP-1 infected with *L*. *donovani* LdBob axenic amastigotes. While 134 hits showed anti-leishmanial activity at 4 µM against Ld1S *L*. *donovani* promastigotes, only 31 hits were confirmed against intramacrophagic amastigotes at 10 µM in our screen (either active, active-toxic or weakly active). We identified 20 hits with high activity against both intracellular *L*. *donovani* and *L*. *amazonensis* amastigotes at 10 µM, five of which showed activity even at 1 µM, thus representing promising candidates for future lead development. Despite various differences between our *ex vivo* and previous *in vitro* screening assays in number of cells seeded per well, incubation time (reduced by 24 hours in our assay), MOI and hit definition, our results open important questions on the impact of host cell and parasite biology on the outcome of phenotypic drug screening efforts and the species- and strain-specific activity we observed for certain anti-leishmanial compounds. Of note, our method uses a model that incorporates physiologically relevant conditions, an important consideration in biological screening campaigns.

Due to technical constraints associated with *ex vivo* material, such as limited access to bulk quantities of bone marrow-derived macrophages or hamster-derived *L*. *donovani* amastigotes, most high throughput screening campaigns were conducted using THP-1 cells and the culture adapted *L*. *donovani* strain LdBob^17,18,23^. Even though this system provides obvious advantages with respect to amount and purity of the *in vitro* generated material, the physiological relevance of both the host cell and the parasite can be questioned, which is the most likely explanation for the low validation rate of Leish-Box hits in our *ex vivo* screening system. THP-1 is a human leukemia monocyte cell line established in the 1980s that can acquire a macrophage-like phenotype after stimulation with phorbol-12-myristate-13-acetate (PMA), 1α, 25-dihydroxyvitamin D3 (vD3) or macrophage colony-stimulating factor (M-CSF)^24,25^. The malignant background of these cells causes important differences in gene expression, immune activation and cellular phenotype compared to bona fide macrophages, and variations in differentiation protocol between laboratories can introduce major bias^26,27^, a concern that is further aggravated by the genomic instability often associated with tumor cell lines that evolve in different lab environments, as recently shown for HeLa cells^28–31^.

Likewise, LdBob parasites^20^ have been cultured over decades and have lost the ability to proliferate inside bona fide macrophages or cause lethal visceral leishmaniasis in the experimental hamster infection^16^, with virulence attenuation resulting from the multiple genomic deletions observed by us and others that affect gp63 and various biopterin transporters^32^ (data not shown) shown to be essential for parasite infectivity^33,34^. This important genomic difference with the parental, virulent Ld1S isolate can be explained by the intrinsic genetic instability of these parasites^13^ and their selection for fitness gains during long-term culture. In addition, this strain underwent several sub-cloning procedures to select for its unique capacity to grow at 37 °C in a host-free (axenic) manner, adopting features similar to amastigotes, such as oval shape or retraction of the flagella^35^. All recently published HT screening results employed these axenic amastigotes as a correlate for bona fide amastigotes either for primary *in vitro* screens on extracellular parasites or for the infection of THP-1 cells to conduct intracellular screens^17,18,23,36^. Even though axenic amastigotes represent an interesting model system to study the parasite response to stress linked to amastigote differentiation^37,38^, the constitutive stress response observed in this purely experimental stage and its profound differences to bona fide amastigotes with respect to transcript output, protein expression, morphology and intracellular proliferation^16,39^ raises major concerns to employ these parasites for drug screening campaigns. Another concern associated with the use of LdBob is its capacity to grow extracellularly at 37 °C^40^. Continuous reinfection during the phenotypic screening procedure would make it impossible to distinguish if reduced intracellular parasite burden is due to direct compound action on intracellular parasites or an indirect action on extracellular growing parasites. This problem has been none the less recently addressed by modifying the THP-1 HTS protocol supplementing the culture medium with horse serum, which has been suggested to eliminate extracellular parasites^41^. Our screening indeed validated most of the Leish-Box hits on extracellular promastigotes but not intracellular amastigotes, an observation that confirms that previous screening results are compromised either by phenotypic limitations of the axenic system, extracellular parasite growth, the lack of proper intracellular parasite development into amastigotes or a combination of all of these aspects.

Nevertheless, despite the low validation rate, our comparative screening approach applied on the Leish-Box hit library yielded interesting hits. First, we identified five compounds that kill both intracellular *L*. *donovani* and *L*. *amazonensis* amastigotes despite the very important differences in the biology of these evolutionary very distant parasites and their interaction with the host macrophage residing in individual or communal parasitophorous vacuoles, respectively. Four of these hits contain nitro-aromatic moieties, as nifurtimox and benznidazole, two drugs used for Chagas’ disease therapy. Those compounds could be pro-drugs activated by parasite nitro-reductases, rendering them toxic. The last hit of the list has chemical similarities to methotrexate and could be an antimetabolite of the antifolate type. Second, a majority of hits were specific to either viscerotropic *L*. *donovani* or dermotropic *L*. *amazonensis*, an observation that is reminiscent to previous studies showing species-specific susceptibility to various reference drugs^42^. In this context, recent data by Hefnawy and colleagues showed the importance to also consider clinical isolates in secondary screens as they revealed different susceptibility profiles to Leish-Box compounds^43^. Less than 50% of the compounds active on LdBOB showed activity against the field isolates. Our screen also revealed a small number of compounds that showed host cell toxicity only in *L*. *donovani* or *L*. *amazonensis* infected BMDMs, demonstrating that differences in species-specific parasite biology and host interaction^44^ need to be considered for the cure of different forms of leishmaniasis as previously suggested^45–49^. Finally, we observed a large number of hits that showed stage-specific activity. Conceivably, promastigote-specific compounds either target stage-specific, essential pathways or fail to cross the various membranes to attain intracellular amastigotes. Likewise, the amastigote-specific activity of various compounds may be explained either by their stage-specific action, or by a requirement for host metabolic processing for their anti-leishmanial action. Given that the Leish-Box hit discovery pipeline included an initial screening step on axenic amastigotes^18^, it seems unlikely that these compounds represent host-directed hits as discussed recently in Lamotte *et al*.^50^. Additionally, the use of promastigotes is considered compulsory in MOA studies of anti-leishmanial hits as it allows to apply biochemical and genetic drug target deconvolution methods for parasite target identification.

In conclusion, both host cells and parasites phenotypes are crucial variables that need to be considered in cell-based screening campaigns. Conceivably, the use of avirulent parasites that fail to establish a productive intracellular infection and the use of immortalized host cells with intrinsic anti-apoptotic properties ultimately can select for false positive hits that fail subsequent validation in pre-clinical models. Physiologically more relevant *ex vivo* screening systems such as presented here, employing virulent amastigotes that proliferate inside bona fide macrophages, can likely overcome this problem. Ultimately, combining these assays with ADMET studies and *in vivo* assays using relevant animal models of leishmaniasis will reduce the very high attrition rate observed for current hit libraries in pre-clinical validation, thus accelerating the lead discovery process. The major challenge for future screening campaigns lies in miniaturizing such *ex vivo* systems, which can only be used by now as confirmatory assays, to render them high through put capable, for example by scaling down the amount of required biological material using microfluidic devices and scaling up the through put using DNA- or peptide-linked compound libraries for combinatorial screening^51,52^.

## Methods

### Animals

Female Golden Syrian hamsters (*Mesocricetus auratus* RjHan:AURA) weighing 60–70 g, six-week-old female BALB/c ByJRj mice and Swiss nu/nu mice were purchased from Janvier Laboratories (Le Genest-Saint-Isle, France), and handled under specific pathogen-free conditions in a A3 animal facilities accredited by the French Ministry of Agriculture for performing experiments on live rodents (agreement A 75-15-01-2). Work on animals was performed in compliance with French and European regulations on care and protection of laboratory animals (EC Directive 2010/63, French Law 2013-118, February 6th, 2013). All experiments were approved by the Ethics Committee “Comité d’éthique en matière d’expérimentation animale de l’Institut Pasteur (#89)” and registered under the reference 2013-0092.

### Parasites

*Leishmania donovani* strain Ld1S (MHOM/SD/*62/1S-CL2D*) and *L*. *amazonensis* strain LV79 (MPRO/BR/1972/M1841) expressing mCherry were propagated in hamsters and Swiss nu/nu mice, respectively. Amastigotes were purified from cutaneous lesions (LV79) and livers (Ld1S) of infected animals^16,19^. Promastigotes were differentiated from animal-derived amastigotes and maintained in modified M199 culture medium as described^16^ but with substantial modifications including absence of detergent and the use of the gentle MACS Dissociator for dissociating infected tissues.

### Mouse primary macrophages

Primary mouse macrophages were prepared as described^19^. Briefly, bone marrow cells were recovered from BALB/c mice and were resuspended in complete DMEM medium (PAN Biotech) supplemented with 75 ng/ml of recombinant mouse CSF-1 (ImmunoTools). Cells were distributed in hydrophobic Petri dishes (Greiner 664161, 1,5 × 10^7^ in 11 ml) and incubated at 37 °C in a 7.5% CO_2_ air atmosphere for 6 days (with mCSF-1 supplemented medium added at day 3).

### Screening procedures

Bone marrow derived macrophages (BMDMs) at day 6 of differentiation were resuspended in complete medium supplemented with CSF-1 (25 ng/ml) and deposited in 384 well plates (culture-treated, flat-optically clear bottom, Cellcarrier plate, PerkinElmer). Five hours later purified Am were added. The cell density and the multiplicity of infection (MOI) were adjusted to the assay conditions as indicated in figure legends.

### Treatment and controls for intracellular amastigote screening

One day after macrophage infection, Leish-Box compounds dissolved in DMSO were added for 3 days at 10 or 1 μM final concentration resulting in a 1% DMSO final concentration. Each compound was tested in quadruplicates. Controls included mock (no liquid added), vehicle control (DMSO), positive control (anti-leishmanial amphotericin B, AmB), and cytotoxicity control (cycloheximide, Cyclo).

### Compound library

Leish-Box contains 192 compounds that have been shown to present anti-leishmanial activity against *L*. *donovani*^18^ but, only 188 were made available for the present study. Compounds are from the 1.8 million GSK HTS screening collection. Distribution of BMDMs, amastigotes and compounds were performed using a robotic platform (Zephyr, Caliper, Perkin Elmer).

### HCS for intracellular *L*. *amazonensis* amastigotes

The high content assay used to assess the anti-leishmanial activity against intramacrophagic amastigotes of *L*. *amazonensis* has been described elsewhere^19^. Briefly, after 3 days of co-incubation with compounds, BMDM nuclei and parasitophorous vacuoles (PVs) were stained with Hoechst 33342 (Invitrogen Molecular Probes, 12 µM) and LysoTracker Green (Life Technologies, DND-26, 1 µM), respectively. Acquisition of images was performed on live cell cultures.

### HCS for intracellular *L*. *donovani* amastigotes

The high content assay used to assess the anti-leishmanial activity against intramacrophagic *L*. *donovani* amastigotes corresponds to the *L*. *amazonensis* HCS protocol up to the drug incubation step. Then, BMDM cultures were fixed with 4% paraformaldehyde solution in PBS for 1 hour at room temperature, washed with PBS, blocked with 50 mM NH_4_Cl-PBS for 15 min, and saturated for 30 min with saponin-PBS (0.1 mg/ml) containing 5% normal goat serum. All washing steps were performed with saponin-PBS. Cells were incubated for 45 min with saponin-PBS with 0.25% gelatin and an immune serum obtained from a *L*. *donovani* infected hamster (dilution 1/1000), washed twice, and further incubated for 45 min with saponin-PBS containing 0.25% gelatin and 2 µg/ml conjugated AlexaFluor 488 goat anti hamster IgG (Jackson ImmunoReasearch). Finally, after additional washing, Hoechst 33342 (5 µg/ml) and HCS CellMask Blue stain (HCS, Invitrogen Molecular Probes, 2 µg/ml) were added for 30 min at room temperature, washed twice with PBS and stored at 4 °C until acquisition. For hit compounds EC50s were calculated using SigmaPlot software (four parameter logistic equation) following application of a range of concentrations (from 10 to 0.16 µM) performed in duplicates.

### Image acquisition and analysis

Images of macrophage cultures were acquired using the automatic Opera QEHS confocal imaging system (Perkin Elmer Technology). For the *L*. *donovani* HCA, a 40x water objective and two lasers (488 nm for immuno-stained parasites; 405 nm for Hoechst-stained parasite and macrophage nuclei, and HCS-stained macrophage cytoplasm) were used for acquiring 285 fields for the entire well or 30 fields per well in routine HCA conditions. For *L*. *amazonensis* HCA, a 10x air objective and three lasers (561 nm for mCherry-expressing parasites; 488 nm for LysoTracker-containing PVs; 405 nm for Hoechst-stained macrophage nuclei) were used to acquire images from entire wells (15 fields).

Images were transferred to the Columbus Conductor™ Database (Perkin Elmer Technologies) and analyzed using the integrated Image analysis building blocks. For *L*. *donovani* HCA, we developed a new script based on object segmentation subroutines detecting successively the macrophage nucleus, the macrophage cytoplasm, the parasites and their nuclei, with their associated features (number, size and object intensity). All methods used for segmentation are described in the Columbus Manuel. Analysis was simplified by only taking into account the number of macrophages and the number of amastigotes per macrophage, which was the common way to estimate the anti-leishmanial activity in screening campaigns. For the sake of clarity, both toxicity against host cells and activity against the parasites of tested compounds were normalized to DMSO-treated controls and expressed as percent of DMSO controls. The viability index is calculated by establishing a ratio of macrophage numbers between compound- and DMSO-treated cells, while the anti-leishmanial activity is represented by the number of amastigotes per macrophage. Compounds causing a 50% or higher reduction of macrophage density were considered toxic. Depending on their parasite viability values expressed as percentages of DMSO control (POC), non-toxic compounds were classified as inactive, weakly active or active for POC above 60%, between 60 and 50% or less than 50%, respectively. Finally, compounds that presented POC values lower than 10% but that also reduced the macrophage density by 40 to 50% were considered as active toxic. Analysis of our biological data showed low standard deviations for all non-toxic, replicate compounds, with standard deviation medians of 9,43% and 15,50% for macrophage and amastigote per macrophage numbers, respectively. For the *L*. *amazonensis* HCA, we applied the method we described previously^19^. Quantitative values obtained for host cell and parasite numbers were exported to Excel and SigmaPlot for further analysis and graphical representations.

### Viability assay for *Leishmania* promastigotes

Anti-leishmanial activity of compounds was evaluated using an adapted resazurin reduction assay^53^ with cell-cycling promastigotes from logarithmic growth phase. Compounds were tested in quadruplicates at 20, 4 and 0.8 μM at 26 °C in 384-well plates. DMSO vehicle and AmB (1 μM) were used as controls. Two days later, resazurin was added (10 μL per well at 25 μg/mL) and fluorescence intensity was measured 24 h later using a Tecan Safire 2 reader (excitation 558 ± 4.5 nm; emission 585 ± 10 nm). Following background subtraction (complete parasite culture medium with resazurin in absence of parasites), data were expressed as percentages of growth compared to DMSO-treated controls (POC).

## Supplementary information

Supplementary information

supplementary table S1
