# Author Correction: Discovery of novel hit compounds with broad activity against visceral and cutaneous *Leishmania* species by comparative phenotypic screening

**DOI:** 10.1038/s41598-019-45360-3

**Published:** 2019-07-09

**Authors:** S. Lamotte, N. Aulner, G. F. Späth, E. Prina

**Affiliations:** 1 Institut Pasteur, Molecular Parasitology and Signaling, INSERM U1201, Department of Parasites and Insect Vectors, Paris, France; 2 Institut Pasteur, UTechS Photonic BioImaging, Center for Technological Research and Resources, 75015 Paris, France

Correction to: *Scientific Reports* 10.1038/s41598-018-36944-6, published online 24 January 2019

A supplementary file containing Table S1 was omitted from the original version of this Article. This has been corrected in the HTML version of the Article; the PDF version was correct at time of publication.

