# Supplementary material for: Discovery of novel hit compounds with broad activity against visceral and cutaneous Leishmania species by comparative phenotypic screening
Source: Sci Rep. 2019 Jan 24;9:438. doi: 10.1038/s41598-018-36944-6 (PMC6345745; doi:10.1038/s41598-018-36944-6)

Discovery of novel hit compounds with broad activity against visceral and cutaneous *Leishmania* species by comparative phenotypic screening

S. Lamotte[1], N. Aulner[2], G.F. Späth[1,*] and E. Prina[1,*]

[1]Institut Pasteur, Molecular Parasitology and Signalisation, INSERM U1201, Department of Parasites and Insect Vectors, [2]Institut Pasteur, UTechS Photonic BioImaging, Center for Technological Research and Resources, 75015 Paris, France.

*co-corresponding authors: Eric Prina, eric.prina@pasteur.fr

Gerald Spaeth, gerald.spaeth@pasteur.fr

Supplementary information

Figure S1. Detection of living intramacrophagic *L. donovani* amastigotes. At three days post-infection, the localization **(A, B)** and the viability of amastigotes **(C, D)** were analyzed by fluorescence. After fixation, BMDMs were immunostained for the lysosome-associated membrane glycoprotein 1 (LAMP1) (rat antibody anti-LAMP1, eBioscience eBio1D4B, 1/100, revealed by secondary antibody anti-rat FITC488, Jackson ImmunoResearch 712-096-153, 10µg/ml) **(A)** or for the parasites (immune serum from a *L. donovani* infected hamster, revealed by secondary antibody anti-hamster AlexaFluor488) **(C)** and stained with Hoechst 33342 and HCS cell mask **(B, D)**. Image acquisition was performed with the OPERA QEHS reader using a 40x water immersion objective (scale bars are 20 µm). Counted amastigotes were always surrounded by a membrane stained with anti-LAMP1 antibodies indicating that amastigotes are strictly intracellular **(A, B)**. Immunostained-amastigotes **(C)** and amastigote nuclei **(D)** in green and blue, respectively. Quantitative analyses were performed in the macrophage cultures without any compound (w/o), in presence of DMSO or AmB (16 wells per condition analyzed, with 20,000 plated BMDMs and a MOI of 6:1). **(E)** As expected, numbers of amastigotes without nuclei (ghost amastigotes) were significantly increased in presence of AmB suggesting an underestimation of anti-leishmanial activity **(F)**.

Figure S2. HCA pipeline. **(A)** Mouse bone marrow cells were cultured in presence of rmCSF-1 during 6 days before BMDM harvesting and plating in 384-well plates. Five and 20 hours later, mouse-derived amastigotes and compounds were added to BMDM cultures, respectively. After 3 days of chemical treatment, LysoTracker Green DND-26 and Hoechst 33342 fluorescent reporters were added to stain parasitophorous vacuoles and macrophage nuclei, respectively, followed by image acquisition on living cells and image analysis. **(B)** HCA readout. Image acquisition was performed on an OPERA QEHS using the green (488 nm), the blue (405 nm) and the red (561 nm) channels for the detection of PV, nuclei and amastigote. **(B1)** Merged green, blue and red fluorescence images acquired with a 10x air objective (NA 0.4) showing the 15 fields of an entire well initially seeded with 20,000 BMDMs and with LV79 mCherry amastigotes at an MOI of 3 (scale bar = 200 µm). **(B2)** Zoomed image corresponding to one acquisition field (scale bar= 100 µm). **(B3)** Cropped and zoomed image

showing fluorescent staining at the cellular level (upper left panel, PV shown in green; upper right panel, Hoechst 33342 staining shown in blue; lower left panel, mCherry expressing amastigotes shown in red; lower right panel, merged images) (scale bar = 40 µm).

Figure S3. Image segmentation and analysis. A representative image of *L. donovani*-infected BMDMs is shown for sequential segmentation steps performed within Columbus™ image analysis platform **(A)**. The script was subdivided into three object segmentation subroutines, detecting successively the nuclei **(B)**, the cytoplasm **(C)** and the intra-cytoplasmic amastigotes **(D)** with their associated features (number, area, roundness and intensity). Validation steps were added to discard false objects using arbitrary-defined threshold set at minimum roundness of 0.55 to detect macrophage nuclei (green objects, **E**), minimum area of 2.1 µm² and minimum roundness of 0.7 to detect parasites (green objects, **F**). **(G)** Optional validation of amastigote integrity was performed by segmenting spots (405 nm channel) inside parasites giving the numbers of living amastigote harboring a nucleus and "ghost" amastigote without nucleus.

Figure S4. Classification of compounds based on biological activity. Compounds were classified according to macrophage viability and number of amastigotes per macrophage expressed as percentages of DMSO control values. Active, macrophage viability > 50% and *Leishmania* survival < 50%; weakly active, macrophage viability > 50% and *Leishmania* survival between 50% and 60%; inactive, macrophage viability > 50% and *Leishmania* survival > 50%; toxic, macrophage viability < 50%; active-toxic, macrophage viability between 40% and 50% and *Leishmania* survival < 10%.

Figure S5. Quality control for HCA quantitative data. A comparative analysis was performed between data obtained from an entire well (285 fields) and data obtained from only 30 fields. **(A)** Well-design showing the distribution of the 30 fields used for the image acquisition during the *L. donovani* HCA with Leish-Box compounds. **(B)** Ratios of estimated macrophage numbers (30 fields) to total macrophage numbers (285 fields) that have been counted in wells seeded with 20,000 or 40,000 BMDMs and treated with DMSO (yellow circles), AmB (green circles) or medium alone

(white circles, w/o). **(C)** Bi-parametric dotplots showing the percentages of infection obtained with 30 fields compared to 285 fields for the different experimental conditions described above. The regression line is shown in black with the determination coefficient R^2.

Figure S6. List of best compounds. Chemical structures of compounds showing strong anti-leishmanial activity against *L. donovani* and *L. amazonensis* intramacrophagic amastigotes.

Supplementary figures

Fig. S1

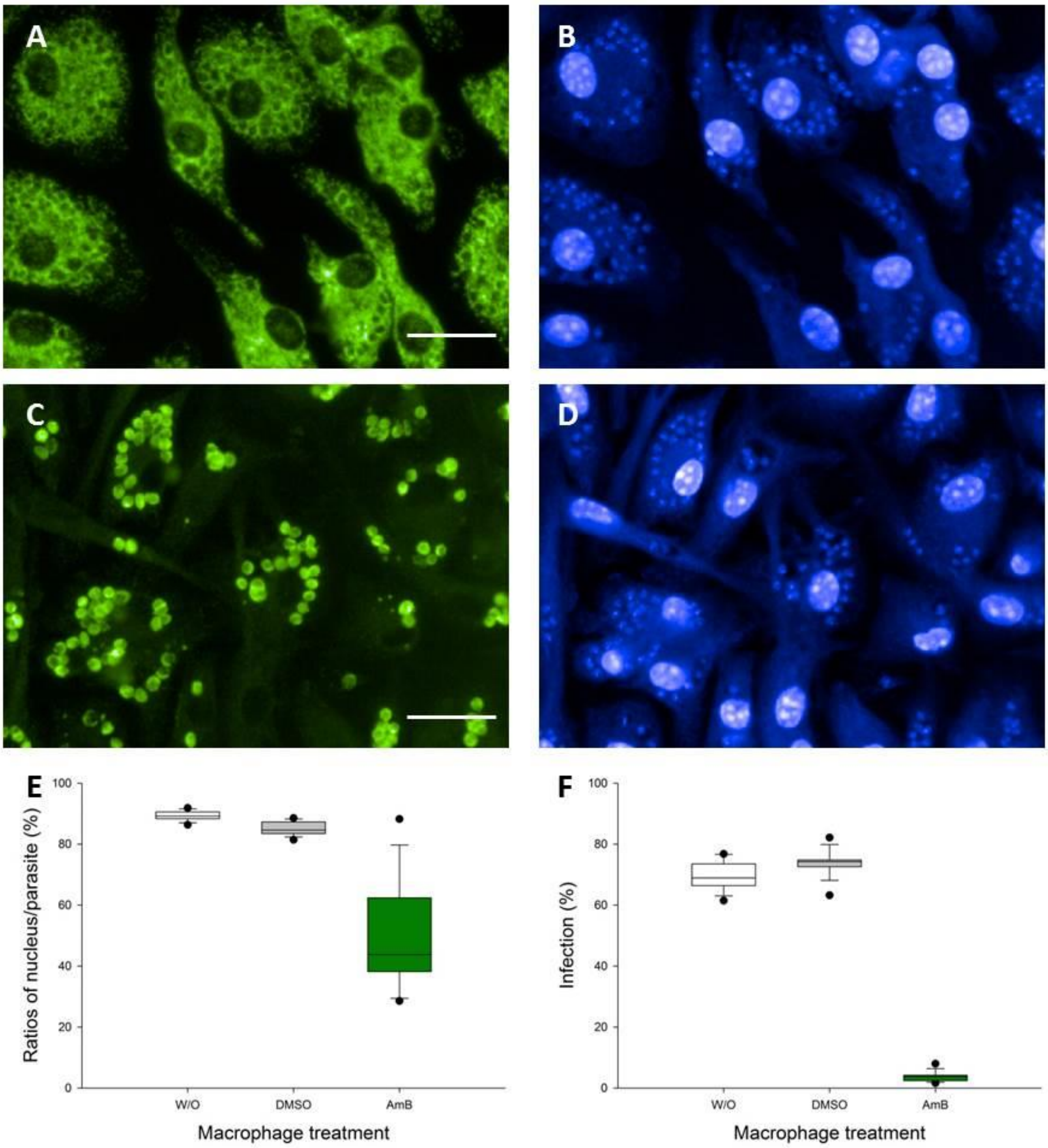

Fig. S2

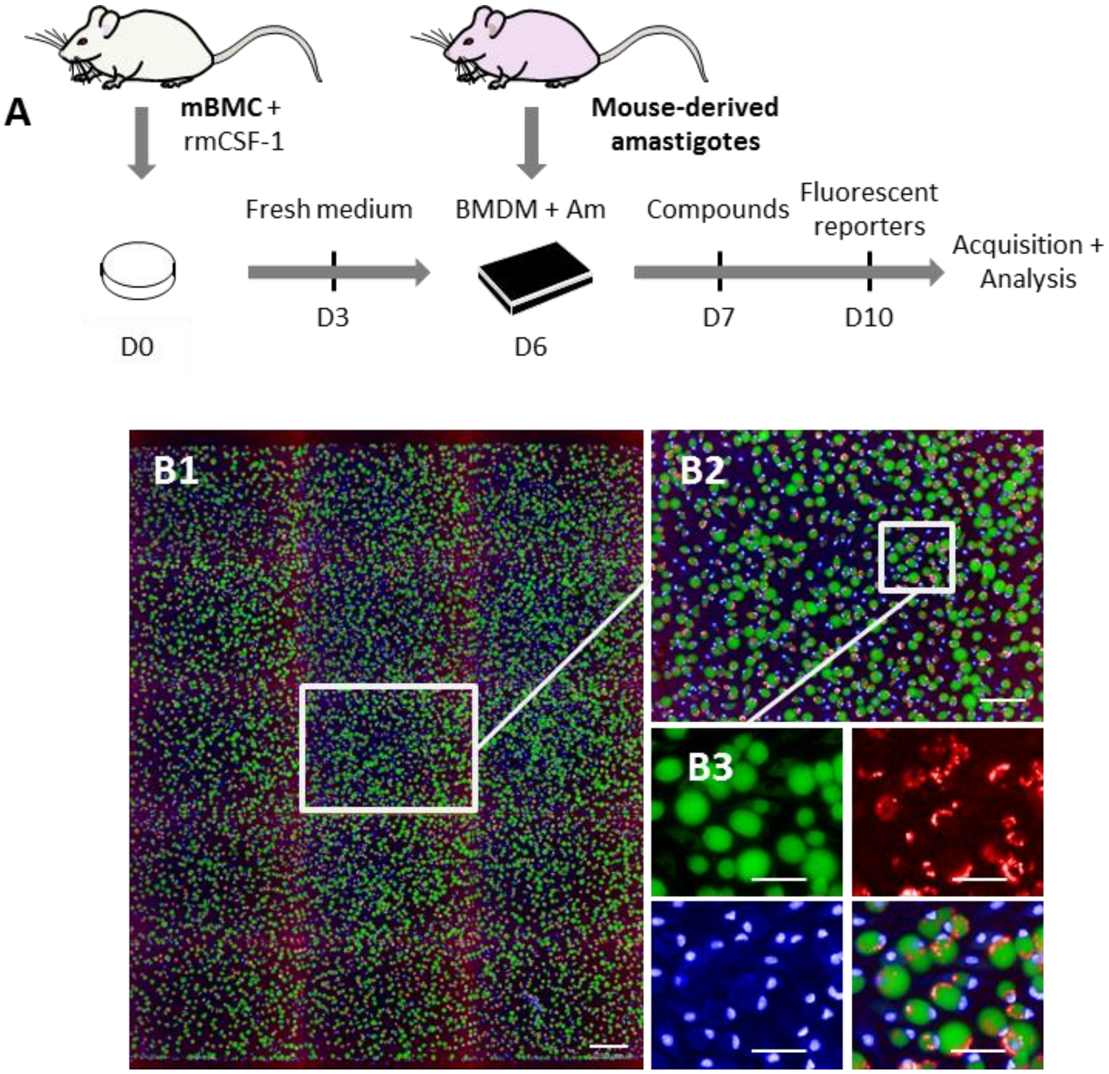
A
mBMC + rmCSF-1
Mouse-derived amastigotes
Fresh medium
BMDM + Am
Compounds
Fluorescent reporters
Acquisition + Analysis
D0
D3
D6
D7
D10
B1
B2
B3

Fig. S3

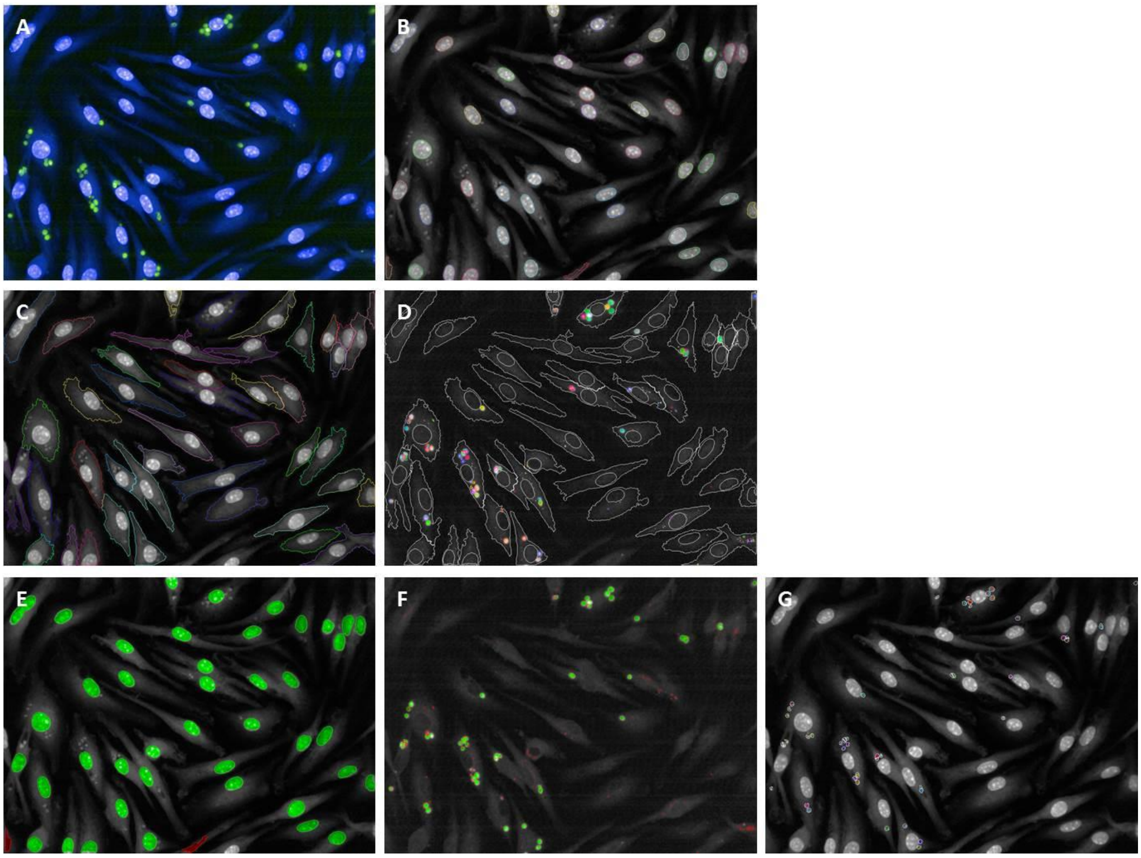
A
B
C
D
E
F
G

Fig. S4

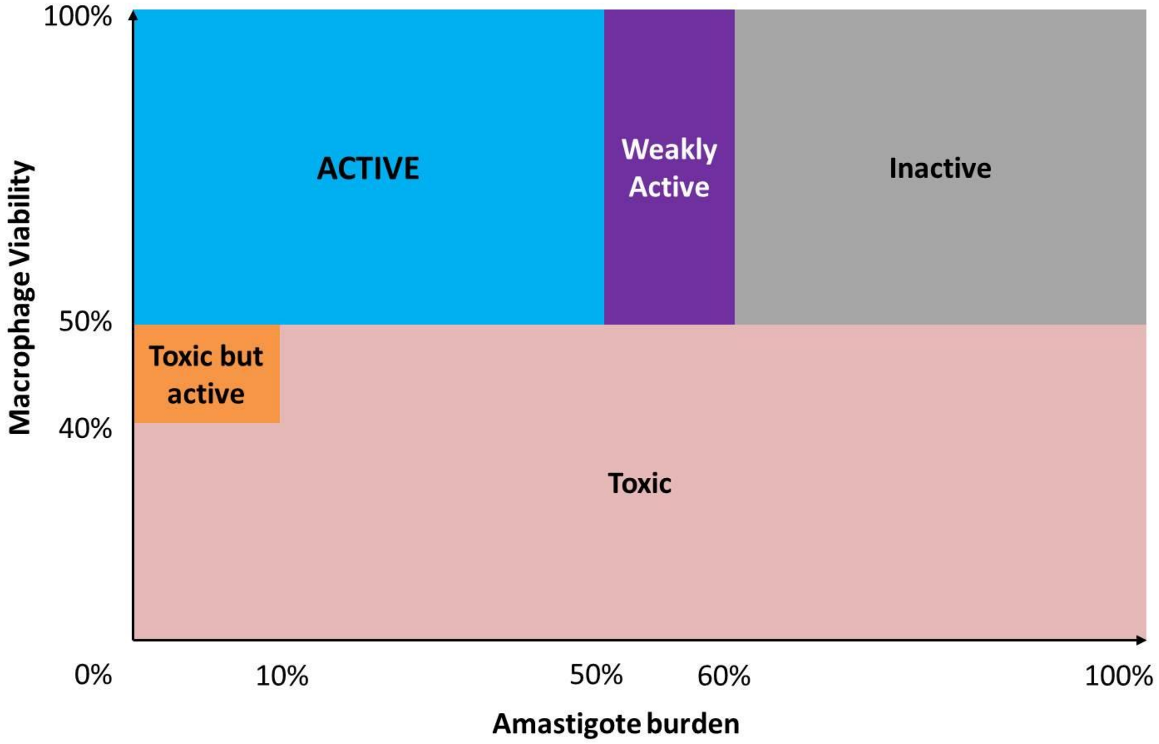
100%
50%
40%
0%
10%
50%
60%
100%
Macrophage Viability
Amastigote burden
ACTIVE
Weakly Active
Inactive
Toxic but active
Toxic

Fig. S5

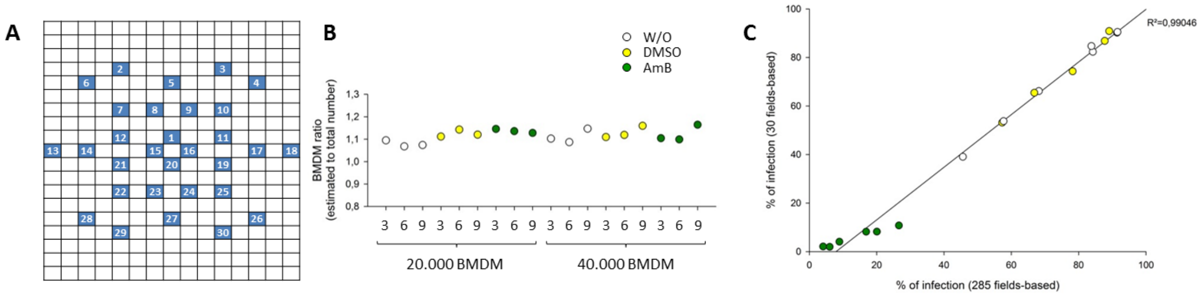

Fig. S6 **Structure of best compounds against *L. donovani and L. amazonensis* amastigotes.**

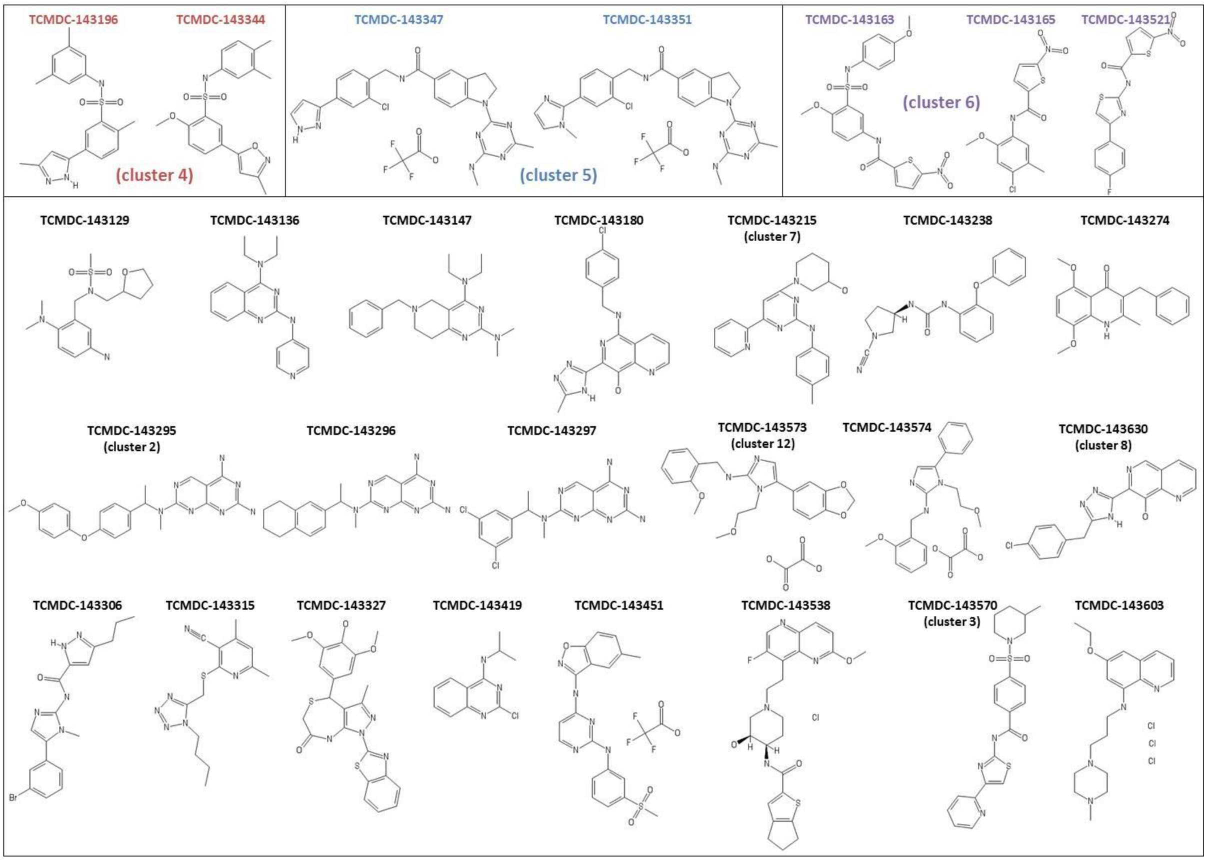

Supplement: Supplementary file 1 — Supplementary information [file 41598_2018_36944_MOESM1_ESM.pdf]
